# Mikronährstoffe im Alter – physiologische Besonderheiten und Versorgungsstatus

**DOI:** 10.1007/s00103-025-04141-2

**Published:** 2025-10-13

**Authors:** Ha Anh Tran, Daniela Weber, Tilman Grune

**Affiliations:** 1 Abteilung Molekulare Toxikologie, Deutsches Institut für Ernährungsforschung Potsdam-Rehbrücke (DIfE), Arthur-Scheunert-Allee 114–116, 14558 Nuthetal, Deutschland; 2 Food4Future (F4F), c/o Leibniz-Institut für Gemüse- und Zierpflanzenbau (IGZ), Großbeeren, Deutschland; 3 Institut für Ernährungswissenschaft, Universität Potsdam, Potsdam, Deutschland

**Keywords:** Ernährung, Mikronährstoffe, Alter, Altersassoziierte Erkrankungen, Vitamine, Nutrition, Micronutrients, Aging, Age-associated diseases, Vitamins

## Abstract

Das Altern ist ein biologischer und degenerativer Prozess, bei dem zahlreiche molekulare und zelluläre Mechanismen in den unterschiedlichsten Organsystemen wirken. Es kommt zu einem fortschreitenden Verlust anatomischer Strukturen und physiologischer Funktionen des Körpers. Die Veränderungen finden u. a. im Herz-Kreislauf-System, Magen-Darm-Trakt, in der Haut, Muskulatur, Knochen, Atemtrakt, Immunsystem, im endokrinen System und im Gehirn statt. Dieser Wandel im Alter kann zu Krankheiten wie koronarer Herzkrankheit, Sarkopenie, Osteoporose, Alzheimer-Demenz und Krebs führen. Um diese altersassoziierten Veränderungen hinauszuzögern und somit auch Krankheiten vorzubeugen, ist ein gesunder Lebensstil mit ausgewogener Ernährung wichtig. Eine adäquate Ernährung umfasst unter anderem die ausreichende Zufuhr von Mikronährstoffen. In diesem Artikel wird zunächst ein Überblick über physiologische Alterungsmerkmale gegeben und im Anschluss auf die Versorgung mit Mikronährstoffen im Alter eingegangen.

Im Alter kann die Versorgung mit Vitamin D, Vitamin B12, Magnesium, Eisen, Folat und Calcium kritisch sein. Diese Mikronährstoffe wirken im Immunsystem, Knochenstoffwechsel, Zellstoffwechsel, in der Energieproduktion und vielen anderen Stoffwechselprozessen im Körper. Ein Mangel kann den Alterungsprozess beschleunigen. Gesunde Ältere können sich an den Referenzwerten der Deutschen Gesellschaft für Ernährung (DGE) orientieren, um ihren täglichen Mikronährstoffbedarf zu decken. Ältere Menschen mit Erkrankungen sollten sich an die Referenzwerte der Leitlinien zu den spezifischen Erkrankungen orientieren, um ihren nötigen Bedarf an Mikronährstoffen zu erreichen.

## Abstract (en)

Aging is a biological and degenerative process with numerous changes in molecular and cellular mechanisms in various organ systems. These changes result in a progressive loss of anatomical structures and physiological functions. Among others, they occur in the cardiovascular system, gastrointestinal tract, skin, muscles, bones, respiratory tract, immune system, endocrine systems, and the brain. The alterations in aging can lead to diseases such as coronary heart disease, sarcopenia, osteoporosis, Alzheimer’s disease, and cancer. To delay these age-related alterations and thus prevent diseases, a healthy lifestyle with a balanced diet is important. An adequate diet includes the sufficient intake of micronutrients. This article first provides an overview of physiological characteristics of aging and then addresses the supply of micronutrients in old age.

In older age, the supply of vitamin D, vitamin B12, magnesium, iron, folate, and calcium may be critical. These micronutrients play a role in the immune system, bone metabolism, cell metabolism, energy production, and many other metabolic processes. Micronutrient deficiencies can enhance the aging process. Healthy older people should use the German Nutrition Society (DGE) reference values to meet their daily micronutrient needs. Older people with illnesses should follow the reference values of the guidelines for their specific illnesses to cover their micronutrient needs.

## Einleitung

Altern ist ein umfassender Prozess, bei dem zahlreiche molekulare und zelluläre Mechanismen in unterschiedlichsten Organsystemen wirken [1]. Ältere Menschen stellen keine homogene Gruppe dar. Je nach Alter, Lebenssituation (selbstständig im eigenen Haushalt oder in einer Einrichtung), Gesundheitszustand, Pflegebedürftigkeit oder Bettlägerigkeit unterscheiden sie sich deutlich in ihrer Ernährung, Nährstoffversorgung und -bedarf. Dieser narrative Übersichtsartikel gibt zunächst einen Überblick über physiologische Alterungsmerkmale und widmet sich anschließend der Mikronährstoffversorgung im höheren Lebensalter. Die dargestellten Aussagen beziehen sich auf bestimmte Kohorten über 65 Jahren und sind nicht uneingeschränkt auf alle Personengruppen dieser Altersklasse übertragbar.

## Wesentliche Alterungsmerkmale und physiologische Veränderungen

Altern als biologischer, degenerativer Prozess umfasst den zunehmenden Verlust physiologischer Funktionen [2]. Der Verlust dieser Funktionen wird auf sogenannte Hallmarks of Aging zurückgeführt. Das sind Merkmale bzw. Kennzeichen der Alterung die den biologischen Alterungsprozess definieren. Allgemein werden 12 Kennzeichen diskutiert (Tab. 1; [3]). Die *Hallmarks of Aging* besitzen eine wechselseitige Abhängigkeit; sowohl die Verstärkung als auch die Abschwächung eines bestimmten Merkmals führt zur Beeinflussung der anderen Merkmale. Altern ist somit ein komplexer Prozess, der als Ganzes betrachtet werden muss [3].

**Tab. 1:** Alterungskennzeichen (Hallmarks of Aging). Eigene Darstellung in Anlehnung an [3]

	Kennzeichen	Erklärung
Primär	Genomische Instabilität	Die Integrität und Stabilität des Genoms werden durch extrinsische und intrinsische Einflüsse geschädigt. Der menschliche Körper hat eine Reihe von DNA-Reparaturmechanismen entwickelt, um diese Schäden auszugleichen und die Stabilität wiederherzustellen. Jedoch verlieren die DNA-Reparaturmechanismen mit dem Alter an Effizienz. Dies führt zu einer Ansammlung von Genomschäden und Instabilität
	Telomerschwund	Telomere sind die Endabschnitte der Chromosomen, die aus sich wiederholenden DNA-Sequenzen bestehen. Sie schützen die Chromosomen vor dem Verlust wichtiger genetischer Informationen. Bei einer Zellteilung können die DNA-Polymerasen die Telomere nicht vollständig replizieren. Die daraus resultierende Verkürzungen der Telomere führt zur Apoptose (programmierter Zelltod) oder Seneszenz (permanenter Stillstand des Zellzyklus)
	Epigenetische Veränderungen	Regulatorische und teilweise reversible epigenetische Veränderungen wirken sich auf die Genexpression und andere zelluläre Prozesse aus. Dabei führen sie zur Entwicklung und zum Fortschreiten verschiedener altersassoziierter Pathologien
	Verlust der Proteostase	Das Altern und altersassoziierte Erkrankungen werden mit einer gestörten Proteinhomöostase assoziiert. Fehlgefaltete Proteine können sich zusammenlagern, was zu einer Anhäufung von geschädigten und nicht funktionsfähigen Proteinen führt
	Gestörte Makroautophagie	Menschliche Zellen sind auf Makroautophagie, einen Prozess zum Abbau beschädigter oder nicht mehr benötigter Zellbestandteile, angewiesen, um ihre Zellfunktionen aufrechtzuerhalten. Ist die Makroautophagie gestört, gerät die zelluläre Homöostase aus dem Gleichgewicht. Die Zellen werden instabil, erleiden Schäden und diese Schäden akkumulieren in Organen und Geweben
Antagonistisch	Fehlregulierte Nährstoffsensorik	Die Nährstoffsensorik sorgt dafür, dass der Körper die richtige Menge an Nährstoffen aufnimmt und den Zustand des Stoffwechselgleichgewichts wieder erreicht. Eine Fehlreaktion der Nährstoffsensoren führt zu Stoffwechselstörungen, die die Alterung beschleunigen können
	Mitochondriale Dysfunktion	Mehrere miteinander verflochtene Mechanismen wie die Ansammlung von mitochondrialen (mt)DNA-Mutationen oder mangelhafte Proteostase führen zu Veränderungen der Dynamik von Mitochondrien. Diese Veränderungen beeinträchtigen den Beitrag der Mitochondrien zur zellulären Bioenergetik, erhöhen die Produktion von Sauerstoffstoffradikalen und lösen möglicherweise eine Permeabilisierung der mitochondrialen Membranen aus, welche Entzündungen und Zelltod verursacht
	Zelluläre Seneszenz	Die zelluläre Seneszenz ist eine Reaktion, die durch akuten Stress oder chronische Anhäufung von Schäden ausgelöst wird. Allgemein wird Seneszenz definiert als permanenter Stillstand des Zellzyklus. Dieser Stillstand führt zu einem irreversiblen Verlust der Replikationsfähigkeit, verbunden mit einer Verringerung spezifischer Zellfunktionen und dem Aufkommen entzündungsfördernder Eigenschaften
Integrativ	Stammzellenerschöpfung	Im Laufe des Lebens sind Stammzellen internem und externem Stress ausgesetzt. Dieser beeinträchtigt ihre genetische Stabilität und Stoffwechselfunktion. Dadurch wird auch ihre allgemeine Funktionalität eingeschränkt, einschließlich der Fähigkeit, Schäden im Körper zu reparieren. Mit der Zeit erschöpfen die Stammzellen, was als eine Hauptursache für die Alterung von Gewebe und des gesamten Organismus gilt
	Veränderte interzelluläre Kommunikation	Das Altern geht mit einer Beeinträchtigung der homöostatischen und hormetischen Regulation (Anpassungsreaktion auf leichte Stressreize) im menschlichen Organismus einher. Dies führt zu Fehlkommunikation in endokrinen, neuronalen und neuroendokrinen Systemen
	Chronische Entzündung	Im Alter kommt es vermehrt zu einer leichten, chronischen Entzündung (Inflammaging), die mit einer Schwächung des Immunsystems einhergeht. Die Entzündungen treten meist nicht isoliert auf, sondern oftmals mit anderen Kennzeichen der Alterung
	Dysbiose	Die Darmflora sendet Signale an das Nervensystem und andere Organe und hat somit einen starken Einfluss auf die allgemeine Erhaltung der Gesundheit des Menschen. Eine Störung dieser Kommunikation führt zu einem Ungleichgewicht der Darmflora

**Abb. 1:**
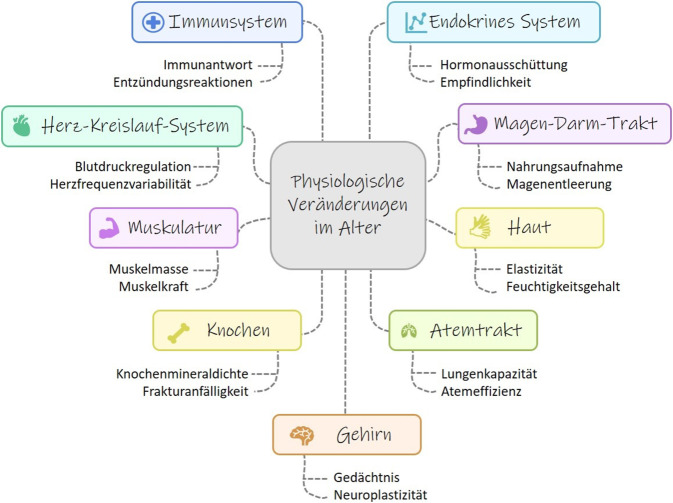
Physiologische Veränderungen im Alter

Die *Hallmarks of Aging* führen zu molekularen Veränderungen im Körper des alternden Menschen. Diese finden sich in unterschiedlichen Organen und Geweben im Körper und beeinflussen das Altern auf unterschiedliche Weise (Abb. 1). Aufgrund des großen Umfangs des Themas kann im Folgenden nur auf die wichtigsten physiologischen Änderungen und ausgewählte Mikronährstoffe eingegangen werden.

### Herz-Kreislauf-System.

Mit zunehmendem Alter verändern sich das Herz und das Gefäßsystem. Das Herz wird größer, verliert aber Herzmuskelzellen, was die Herzfunktion beeinträchtigen kann. Gleichzeitig werden die Blutgefäße steifer [4]. Diese Veränderungen, einschließlich einer beeinträchtigten Endothelzellfunktion, führen zu einem erhöhten Risiko für Gefäßerkrankungen, Blutgerinnung und einem erhöhten Blutdruck [4]. Letztendlich können diese Veränderungen zu Erkrankungen wie Herzinfarkt, koronarer Herzkrankheit und Herzinsuffizienz führen [5].

### Magen-Darm-Trakt.

Eine normale Funktion des Gastrointestinaltrakts ist grundlegend für die Aufnahme von Nährstoffen und Medikamenten im Körper. Darüber hinaus schützt ein intakter Magen-Darm-Trakt den Menschen vor aufgenommenen Krankheitserregern. Im Mund, in der Speiseröhre, im Magen sowie im Dünn- und Dickdarm greifen vielfältige Mechanismen, die die Verdauung und Aufnahme von Nährstoffen steuern. Mit zunehmendem Alter können diese Prozesse beeinträchtigt sein. So treten Schluckstörungen häufiger auf und erschweren die Nahrungs- und Nährstoffaufnahme [6]. Zudem kommt es im Alter vermehrt zu Zahnverlust, wodurch Kauen und Zerkleinern der Nahrung erschwert werden, was wiederum das Risiko einer Mangelernährung erhöht [7].

Das Altern ist mit unterschiedlichen Veränderungen der gastrointestinalen Muskulatur assoziiert. Diese wirken sich auf die Motilität aus und betreffen unter anderem die Peristaltik der Speiseröhre, die Magenentleerung sowie die Passagezeiten im Dickdarm. Solche Veränderungen können zu einem frühen Sättigungsgefühl, verringerter Nahrungsaufnahme und in der Folge zu Gewichtsverlust und Nährstoffmangel führen [8]. Ältere Menschen vermeiden häufig fettreiche Nahrung, da diese länger im Magen verbleibt und Sodbrennen verursachen kann. Zudem kann die Verdaulichkeit von fettigen Speisen reduziert sein, da sich die Zusammensetzung der Galle im Alter verändert und die Kontraktionsfähigkeit der Gallenblase abnimmt. Es ist jedoch unklar, ob die Gallenproduktion abnimmt. Das Vermeiden von fettreichen Lebensmitteln könnte eine Reduktion der Absorption von fettlöslichen Vitaminen begünstigen. Weiterhin nimmt die Anzahl der Nervenzellen ab, die für die Regulation von Verdauung und Absorption zuständig sind [6].

Auch lässt die Immunfunktion mit dem Alter nach. Bakterielle und virale Infektionen des Magen-Darm-Trakts treten bei älteren Menschen häufiger auf [9]. Zudem kommt es zur Modifikation des Mikrobioms. Die mikrobielle Vielfalt im Darm nimmt mit dem Alter ab. Die Häufigkeit verschiedener Bakterien wie *Bacteroides* und *Clostridium* ist verändert und kann zu ungesunder Mikroflora oder Dysbiose führen [10]. Ein höheres Alter ist mit strukturellen und funktionellen Defekten der Schleimhautabwehr, erhöhtem oxidativen Stress, einer verminderten Fähigkeit zur Ausbildung einer schützenden Immunität sowie mit einer erhöhten Inzidenz von Entzündungen und Autoimmunität assoziiert [6]. Allgemein sind ältere Menschen besonders anfällig für Mangelernährung, postprandiale Hypotonie, Dysphagie, Verstopfung und Stuhlinkontinenz [6].

### Haut.

Die Haut dient als mechanisch schützende und flexible Barriere. Mit dem Alter verändert sie sich und ist durch einen fortschreitenden Verlust an Funktionalität und Regenerationspotenzial gekennzeichnet. Der Hautalterungsprozess wird durch intrinsische und extrinsische Faktoren beeinflusst. Intrinsische, chronologische Hautalterung ist ein unvermeidbarer Vorgang chronologischer und physiologischer Transformationen. Zu den intrinsischen Faktoren gehören Zeit, Genetik und Hormone. Die intrinsische Hautalterung zeigt sich in Form von feinen Linien, Trockenheit und Schlaffheit. Die extrinsische Hautalterung betrifft meist exponierte Bereiche wie Gesicht, Hals und Hände. Zu den extrinsischen Faktoren zählen Sonnenlicht, Luftverschmutzung, Zigarettenrauch, Ernährungsfaktoren, Temperatur, Stress und Schlafmangel. Die extrinsische Alterung äußert sich in Form von groben Falten und Pigmentveränderungen wie unregelmäßiger Pigmentierung und Altersflecken [11, 12].

Bei älteren Männern findet eine Umverteilung des Fettgewebes vom subkutanen hin zum viszeralen Fettdepot statt; bei Frauen konnte dies nicht nachgewiesen werden [13]. Welche Folgen dies hat, wurde bislang unzureichend untersucht, jedoch könnte eine Auswirkung auf die Vitamin-D-Produktion bestehen, welche im Alter per se verringert ist [14].

### Muskulatur.

Mit fortschreitendem Alter ändert sich der Muskelmetabolismus. Es kommt zu einem altersassoziierten Muskelschwund, der Sarkopenie [15]. Die verringerte Anzahl der motorischen Einheiten, die geringe Beanspruchung und nachlassende Durchblutung der Muskeln führen zum Abbau von Muskulatur. Die Histologie alternder Muskeln zeigt eine Heterogenität in der Fasergröße, eine vermehrte Vernetzung von Muskelfasern und einen Verlust an Satellitenzellen auf [16].

Der fortschreitende Verlust der Skelettmuskulatur wirkt sich negativ auf mehrere physiologischen Parameter aus; dazu gehören Bewegung, Atmung, Sehvermögen, Wärmeregulierung und metabolische Homöostase [17]. Die altersassoziierten muskulären Veränderungen stehen oftmals im Zusammenhang mit Gebrechlichkeit (Frailty). Frailty bezeichnet einen Zustand verminderter Fähigkeit, auf physische und psychische Belastungen adäquat zu reagieren. Damit geht ein erhöhtes Risiko für Stürze, Behinderungen und Krankenhausaufenthalte einher [16].

### Knochen.

Ein natürlicher physiologischer Prozess, der mit dem Alter einhergeht, sind die Abnahme der Knochendichte, eine Schwächung der inneren Knochenstruktur und eine erhöhte Anfälligkeit für Schäden am Skelettsystem. Diese werden durch Stoffwechselprozesse wie erhöhte Knochenresorption, verringerte Knochenbildung und die Störung wichtiger Signalwege im Knochenmetabolismus verursacht. Alternde Menschen sind deshalb anfälliger für Frakturen und Knochenerkrankungen wie Osteoporose [18].

### Atemtrakt.

Mit dem Alter verändern sich sowohl die inneren als auch die stützenden Strukturen der Lunge [19]. Charakteristisch sind eine abnehmende Bronchiolendichte und ein vergrößerter Bronchiolendurchmesser. Zudem kommt es zu einem Verlust der Alveolaroberfläche, verbunden mit einem vergrößerten Alveolar- und Luftraum. Die Lunge verliert ihre Elastizität und wird starrer [20]. Diese strukturellen Veränderungen führen zu einem weniger effizienten Atemmechanismus. Es kommt zu einer verringerten Ausatmung und gleichzeitig zu einem erhöhten Lufteinschluss sowie einem vergrößerten Volumen der eingeschlossenen Luft. Die Folge ist ein verringerter Gasaustausch in den Lungen [19]. Fortgeschrittenes Alter ist ein Hauptrisikofaktor für die Entwicklung von chronischen Lungenerkrankungen wie der chronisch obstruktiven Lungenerkrankung, interstitiellen fibrotischen Lungenerkrankung oder auch Lungenkrebs [20].

### Immunsystem.

Im Alter lässt die Immunfunktion nach; ältere Menschen reagieren nicht mehr effizient auf neue oder bereits vorhandene Antigene. Das Immunsystem älterer Menschen reagiert weniger durch naive Immunzellen, sondern vermehrt über dysfunktionale Gedächtniszellen. Weiterhin kommt es zur Rückbildung der primären lymphatischen Organe und zu einer veränderten Immunantwort des angeborenen Immunsystems. Dies führt zu einer höheren Anfälligkeit für Infektionskrankheiten und einer verringerten Reaktion auf Impfungen [21]. Zudem weisen ältere Menschen eine leichte, aber chronische systemische Entzündung (Inflammaging) sowie eine erhöhte Inzidenz von Autoimmunität und eine beeinträchtigte Wundheilung auf. All diese altersassoziierten Prozesse im Immunsystem werden allgemein als „Immunseneszenz“ bezeichnet [1]. Insgesamt ist die ältere Bevölkerung aufgrund dieser Immunseneszenz anfälliger für Infektionen, insbesondere für Influenza, Pneumokokken- und Streptokokkeninfektionen, aber auch für wiederkehrende chronische Infektionen wie Herpesvirus-Infektionen, etwa Gürtelrose [21].

Obwohl allergische Erkrankungen traditionell vor allem bei jüngeren Menschen auftreten, werden sie zunehmend auch im höheren Alter beobachtet – etwa 5–10 % der älteren Bevölkerung sind betroffen und aufgrund altersassoziierter Veränderungen des Immunsystems ist ein weiterer Anstieg zu erwarten [22].

### Endokrines System.

Altern ist mit einer Zunahme von Störungen im endokrinen System assoziiert. Einerseits betrifft dies die Hormonausschüttung in der endokrinen Achse, andererseits nimmt die Empfindlichkeit der Achse gegenüber Zielhormonen ab, ebenso wie die Empfindlichkeit der Zielgewebe gegenüber diesen Hormonen. Endokrine Dysfunktionen beeinflussen den Metabolismus ubiquitär; sie gehen mit Störungen der Glukosehomöostase, dem Verlust von Muskel- und Knochenmasse sowie Autoimmun- und degenerativen Erkrankungen einher [23].

### Gehirn.

Die Morphologie des Gehirns ändert sich mit dem Alter, was sich vor allem in Form einer Atrophie zeigt. Charakteristisch sind eine Abnahme des Gehirnvolumens, eine Ausdünnung der Hirnrinde, ein Abbau der weißen Substanz, ein Verlust von Neuronen sowie eine verminderte Gyrifikation (typische gefurchte, wellenförmige Oberflächenstruktur der Hirnrinde). Weiterhin weist das alternde Gehirn auch pathophysiologische Modifikationen auf; dazu gehören der Verlust einzelner Neuronenpopulationen, dendritische Degeneration, Kleingefäßerkrankungen und Läsionen der weißen Substanz. Zudem kann es zu einer fortschreitenden Ansammlung von Proteinen und Peptiden kommen, die sich u. a. als Amyloid-Plaques im Gehirn ablagern. Aufgrund dieser morphologischen und pathophysiologischen Veränderungen kommt es zu kognitiven Defiziten, Gedächtnisverlust, verminderter motorischer Leistungsfähigkeit und Änderungen im Verhalten. Eine starke Ausprägung ist ein Anzeichen für eine beginnende Alzheimer-Demenz [24].

## Mikronährstoffversorgung im Alter

### Referenzwerte der DGE für die Mikronährstoffzufuhr bei Älteren

Die Referenzwerte der Deutschen Gesellschaft für Ernährung (DGE) für die Mikronährstoffzufuhr bei älteren Erwachsenen (ab 65 Jahren) unterscheiden sich kaum von den Referenzwerten für Erwachsene unter 65 Jahren (Tab. 2; [25]). Die kürzlich verabschiedeten lebensmittelbezogenen Ernährungsempfehlungen beziehen sich nur auf gesunde Erwachsene von 18 bis 65 Jahren [26]. Da sich der Körper, wie oben beschrieben, mit zunehmendem Alter verändert und altersassoziierte Erkrankungen auftreten können, verändert sich jedoch auch der Mikronährstoffbedarf. Ältere Menschen mit Erkrankungen sollten sich deshalb an den Referenzwerten der jeweiligen krankheitsspezifischen Leitlinien orientieren.

**Tab. 2:** Referenzwerte der Deutschen Gesellschaft für Ernährung (DGE) für ältere Erwachsene ab 65 Jahre. Datenquelle: [25]

Mikronährstoffgruppe	Mikronährstoff	m	w	Vorkommen
Fettlösliche Vitamine	Vitamin A [µg/d]^a^	800	700	Leber, Karotten, Kürbis, grünes Blattgemüse
	Vitamin D [µg/d]	20		Fettfische, Eier, einige Speisepilze
	Vitamin E [mg/d]^a^	12	11	Nüsse, Samen, grünes Blattgemüse
	Vitamin K [µg/d]	80	65	Grünes Gemüse, Milch, Fleisch, Eier
Wasserlösliche Vitamine	Ascorbinsäure (Vitamin C) [mg/d]	110	95	Zitrusfrüchte, Tomaten, Kartoffeln
	Thiamin (Vitamin B_1_) [mg/d]	1,1	1,0	Vollkornprodukte, Samen, Hülsenfrüchte
	Riboflavin (Vitamin B_2_) [mg/d]	1,3	1,0	Leber, Niere, Fisch, Milch, Milchprodukte
	Niacin (Vitamin B_3_) [mg/d]^a^	14	11	Fisch, mageres Fleisch, Brot, Erdnüsse, Pilze
	Pantothensäure (Vitamin B_5_) [mg/d]	5		Fleisch, Fisch, Eier, Pilze, Vollkornprodukte
	Pyridoxin (Vitamin B_6_) [mg/d]	1,6	1,4	Vollkorngetreide, Walnuss, Sardinen
	Biotin (Vitamin B_7_) [mg/d]	40		Innereien, Sojabohnen, Nüsse, Pilze, Milch
	Folsäure (Vitamin B_9_) [µg/d]^a^	300		Grünes Blattgemüse, Hülsenfrüchte, Nüsse
	Cobalamin (Vitamin B_12_) [µg/d]	4,0		Milch, Eier, Fisch, Meeresfrüchte, Geflügel
Mineralstoffe (Mengenelemente)	Natrium [mg/d]	1500		Brot, Käse, Wurstwaren, Fischkonserven
	Chlorid [mg/d]	2300		Speisesalz, Brot, Käse, Wurstwaren
	Kalium [mg/d]	4000		Aprikose, Bananen, Karotten, Kohlrabi
	Calcium [mg/d]	1000		Grünes Gemüse, Samen, Nüsse
	Phosphor [mg/d]	550		Hülsenfrüchte, Nüsse, Samen, Fleisch
	Magnesium [mg/d]	350	300	Vollkornprodukte, grünes Blattgemüse, Nüsse
Mineralstoffe (Spurenelemente)	Eisen [mg/d]	11	14	Vollkornprodukte, Fleisch, Hülsenfrüchte
	Jod [µg/d]	180		Seefisch, Milch, Eier, jodiertes Speisesalz
	Fluorid [mg/d]	3,5	3,0	Fluoridiertes Speisesalz, Fische, Schwarztee
	Zink [mg/d]	14	8	Fleisch, Eier, Milchprodukte, Hülsenfrüchte
	Selen [µg/d]	70	60	Kohlgemüse, Zwiebelgemüse, Pilze, Spargel
	Kupfer [mg/d]	1,0–1,5		Getreideprodukte, Nüsse, grünes Gemüse
	Mangan [mg/d]	2,0–5,0		Grünes Gemüse, Haferflocken, Tee
	Chrom [µg/d]	30–100		Fleisch, Eier, Haferflocken
	Molybdän [µg/d]	50–100		Hülsenfrüchte, Getreide

*m* männlich, *w* weiblich

^a^ µg bzw. mg Vitamin-Äquivalente

Der Mikronährstoffbedarf sowohl gesunder als auch erkrankter älterer Menschen ist bislang nicht ausreichend untersucht, sodass die in den Leitlinien angegebenen Referenzwerte lediglich als grobe Orientierung dienen können. Um die optimale Mikronährstoffzufuhr bei Älteren zu erreichen, ist weitere Forschung notwendig, auch mit Ansätzen der personalisierten Ernährung und stratifizierten Ernährungsinterventionen.

### Einflussfaktoren auf die Mikronährstoffzufuhr im Alter

Die Nahrungszufuhr bei älteren Erwachsenen wird durch zahlreiche intrinsische und extrinsische Faktoren beeinflusst wie etwa physiologische und pathophysiologische Prozesse (siehe oben) sowie Zugang zu Nahrung, Beziehung zu Nahrung, sozioökonomischer Status (Bildung, Beruf, Einkommen), soziale Gewohnheiten und soziale Isolation. Psychologische Faktoren bei Älteren, die oft mit Appetitlosigkeit oder einer Veränderung der Nahrungsaufnahme einhergehen, sind Depressionen, Demenz oder kognitive Beeinträchtigungen. Auch Medikamente können zu einem schlechten Mikronährstoffstatus führen, da sie die Bioverfügbarkeit der Stoffe beeinflussen können. All diese Faktoren tragen dazu bei, dass ältere Menschen die empfohlenen täglichen Mikronährstoffmengen seltener erreichen und dadurch ein Risiko für Mangelernährung besteht. Diese wiederum ist im Alter mit einem Verlust der Fähigkeit zur Ausführung alltäglicher Aktivitäten verbunden [27].

### Besonderheiten der Versorgung mit Mikronährstoffen im Alter

**Abb. 2:**
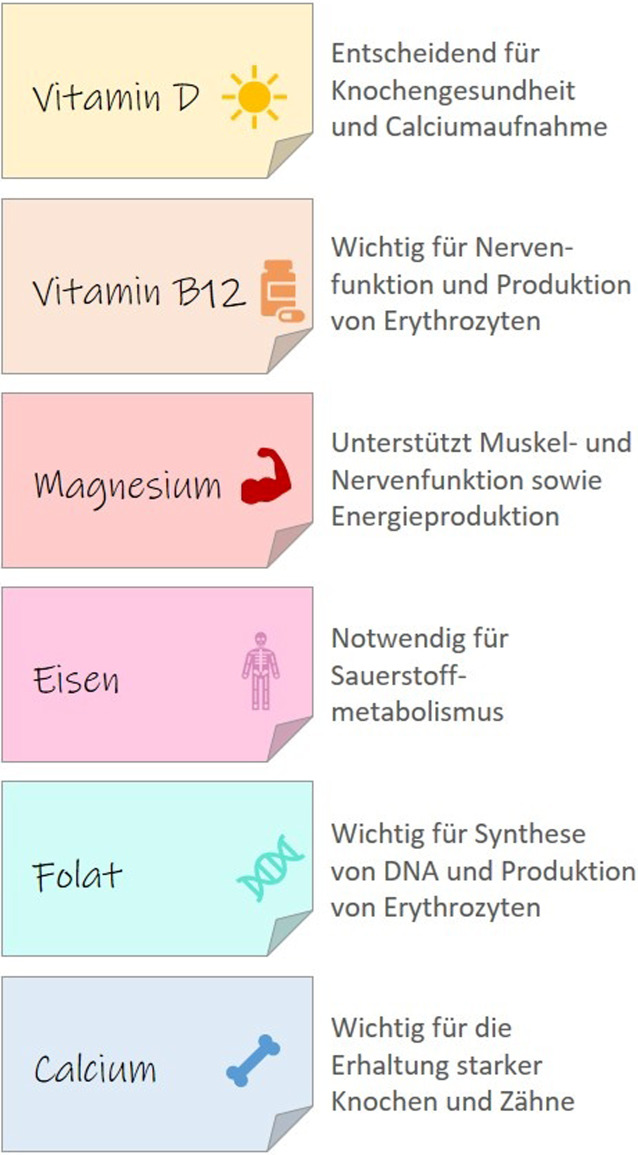
Bedeutung von Mikronährstoffen im Alter

Um altersassoziierte Veränderungen hinauszuzögern und Krankheiten vorzubeugen, ist ein bewusster Lebensstil mit einer gesunden, ausgewogenen Ernährung wichtig. Eine unzureichende Zufuhr von Mikronährstoffen kann erhebliche negative Folgen für die Gesundheit haben [28]. Im Folgenden werden einige – in der Versorgung besonders kritische – Mikronährstoffe näher betrachtet (Abb. 2). Der Fokus liegt auf Vitamin D, Vitamin B12 und Magnesium. Eisen, Folat und Calcium werden ergänzend kurz behandelt, da sie – ähnlich wie auch in anderen Bevölkerungsgruppen – in der Versorgung kritisch sein können.

#### Vitamin D.

Das Hormon Vitamin D wird in der Ernährung hauptsächlich in Form von Cholecalciferol (Vitamin D3) und Ergocalciferol (Vitamin D2) zugeführt. Cholecalciferol ist in Lebensmitteln tierischen Ursprungs, Ergocalciferol in pflanzlichen Lebensmitteln enthalten. Die Aufnahme von Vitamin D über die Nahrung ist sehr gering, da die Auswahl an Vitamin-D-haltigen Lebensmittelquellen beschränkt ist. Jedoch kann das Vitamin auch durch die ultravioletten Strahlungen der Sonne in der Haut synthetisiert werden.

Die wichtigsten Wirkungsorte von Vitamin D befinden sich in Geweben wie Knochen, Darm und Niere. Dort wirkt das Vitamin in der Calciumhomöostase und der Knochenmineralisierung. Weiterhin reguliert Vitamin D das angeborene und das adaptive Immunsystem und hemmt dadurch z. B. überschießende Immunreaktionen. Darüber hinaus spielt Vitamin D eine Rolle bei der Zellregeneration im Darm, im Herz-Kreislauf-System, in den Muskeln und im Gehirn. Ein Defizit des Vitamins führt zu Muskelatrophie, Gebrechlichkeit, verminderter Knochenbildung, erhöhtem oxidativen Stress und Entzündungen. Ein Mangel ist mit dem Auftreten von Knochenveränderungen, Herz-Kreislauf-Erkrankungen, dem metabolischen Syndrom, Immunerkrankungen und Sarkopenie assoziiert [29].

Ältere Menschen sind häufig von Vitamin-D-Mangel betroffen, da sie sich weniger dem Sonnenlicht aussetzen. Eine aktuelle Studie aus Deutschland an älteren Personen mit Frakturen belegt, dass weder die körpereigene Synthese noch die Zufuhr über die Nahrung ausreicht, um den Bedarf zu decken, sodass eine Supplementierung notwendig ist [30]. Weiterhin könnte eine Malabsorption im Darm zu verminderter Aufnahme des Vitamins bei Älteren führen. In Deutschland erreichen 94 % der Männer und 97 % der Frauen über 65 Jahren nicht die täglich empfohlene Zufuhr an Vitamin D, dies trifft jedoch ebenfalls auf jüngere Altersgruppen zu [31]. In der KORA-Studie wurde als Referenzwert 50 nmol/l zugrunde gelegt, hier zeigten 52 % der Teilnehmenden einen subklinischen Vitamin-D-Mangel [32].

Zu beachten gilt, dass verschiedene Fachgesellschaften unterschiedliche Referenzwerte verwenden, was sowohl die Vergleichbarkeit als auch die Einstufung in Vitamin-D-Statusklassen erschwert. Die vom Institute of Medicine (IOM) und vom Robert Koch-Institut (RKI) befürworteten Referenzwerte besagen, dass ein 25(OH)D-Spiegel (25-Hydroxyvitamin D_3_, Transport- und Speicherform von Vitamin D) zwischen 30 nmol/l und 50 nmol/l als „suboptimale Versorgung mit möglichen Folgen für die Knochengesundheit“ und erst unter 30 nmol/l als „Mangelhafte Versorgung mit einem erhöhten Risiko für Krankheiten wie Rachitis, Osteomalazie und Osteoporose“ einzustufen ist. Die Endocrine Society hingegen nahm kürzlich ihre Referenzwerte (> 75 nmol/l) für eine ausreichende Versorgung zurück, da die verfügbaren Studienergebnisse nicht eindeutig zeigen, dass bei grundsätzlich gesunden Personen ein Netto-Nutzen durch Vitamin-D-Supplementierung spezifisch durch 25(OH)D-Konzentrationen unter 50–60 nmol/l vorhergesagt wird [33].

Aufgrund der unzureichenden oralen Zufuhr und endogenen Synthese des Vitamins bei älteren Individuen, ist der Verzehr von Vitamin-D-Präparaten vorteilhaft, um den täglichen Vitamin-D-Bedarf zu decken [30, 34]. Die Hauptquelle für die Vitamin-D-Zufuhr aus der Nahrung in Deutschland ist Fisch [31]. Aufgrund des geringen Gehaltes von Vitamin D in Lebensmitteln ist die Deckung des Bedarfs von 20 µg/Tag über die Nahrung alleine nicht ausreichend, über eine häufige Sonnenexposition jedoch möglich [35].

#### Vitamin B12.

Cobalamin (Vitamin B12) umfasst eine Gruppe verschiedener kobalthaltiger Verbindungen. Zu diesen Verbindungen gehören Methylcobalamin und Desoxyadenosylcobalamin. Sie stellen metabolisch aktive Formen des Vitamins B12 dar. Die Cobalaminzufuhr erfolgt aus Lebensmitteln tierischen Ursprungs wie Fleisch, Eier, Fisch und Milchprodukten. Vitamin B12 fungiert als Co-Faktor für 2 Enzyme: Methioninsynthase und Methylmalonyl-CoA-Mutase. Diese Enzyme wirken in wichtigen Reaktionen verschiedener Stoffwechselprozesse. Die Methioninsynthase katalysiert die Umwandlung von Homocystein zu Methionin. Diese Reaktion spielt eine zentrale Rolle bei der Regulierung des Homocysteinspiegels im Körper und beeinflusst die Methylierung von Phospholipiden, Neurotransmittern, Purinbasen, DNA, RNA und basischen Myelinproteinen. Die Methylmalonyl-CoA-Mutase katalysiert die Umwandlung von Methylmalonyl-CoA zu Succinyl-CoA. Diese Reaktion ist ein wichtiger Schritt im Stoffwechsel und spielt eine Rolle bei der Energieproduktion [36].

Die Absorption von Vitamin B12 wird mit dem Alter weniger effizient, aufgrund altersassoziierter Veränderungen, z. B. aufgrund einer *Helicobacter-pylori-*Besiedlung. Vitamin B12 benötigt im Magen einen *Intrinsic Factor *(IF) zur Stabilisierung gegenüber der Magensäure. So bildet Vitamin B12 mit dem IF einen Komplex, welcher im Ileum absorbiert werden kann. Bei Gastritis wird dieser IF oft nicht ausreichend gebildet. Ältere Menschen neigen eher dazu eine (chronische) Gastritis zu entwickeln [6, 36]. Die Malabsorption kann nahrungsbedingt sein, aber auch durch Wechselwirkungen mit Medikamenten entstehen. Eine Malabsorption, aber auch die geringe Aufnahme von Vitamin B12 oder Autoimmunerkrankungen können zu einem Mangel im Alter führen [36, 37].

Ein Vitamin-B12-Defizit hat hämatologische, neurologische und neuropsychiatrische Auswirkungen. Im Alter häufig auftretende Krankheiten wie kardiovaskuläre Erkrankungen, Schlaganfall, Depressionen, Alzheimer, Osteoporose und Frakturen werden mit Vitamin-B12-Mangel assoziiert [36, 37]. Interventionsstudien untersuchen meist die Kombination verschiedener B‑Vitamine, sodass die isolierte Wirkung von Vitamin B12 schwer abzuschätzen ist. Die mechanistischen Zusammenhänge zwischen Vitamin B12 und Alzheimer werden bei Lauer et al. ausführlich beschrieben [38].

Eine aktuelle Studie aus Deutschland mit gesunden, zuhause lebenden Senioren (≥ 70 Jahre) fand eine Vitamin-B12-Mangel-Prävalenz von 12 % (unter allen Teilnehmenden) bzw. 22 % (unter Männern; [39]), einen subklinischen Mangel wiesen 27,3 % der Teilnehmenden der KORA-Studie auf [32]. In Deutschland erreichen 10 % der älteren Männer und 26 % der älteren Frauen nicht den täglichen Schätzwert für eine angemessene Zufuhr an Vitamin B12 [31]. Um den täglichen Bedarf des Vitamins zu erreichen, ist beispielsweise ein Verzehr von einem Glas Milch, einem Becher Joghurt, einem Ei und 60 g Camembert am Tag nötig [40].

#### Magnesium.

Der Mineralstoff Magnesium ist für zahlreiche biologische Prozesse im Körper unverzichtbar ist. So ist Magnesium ein Teil des Energiestoffwechsels; dort ist es an der mitochondrialen Synthese von Adenosintriphosphat (ATP) beteiligt. Außerdem spielt Magnesium eine Rolle bei der Übertragung von neuronalen Signalen und bei der Muskelkontraktion. Weiterhin hilft es bei der Regulierung des Blutdrucks und ist beteiligt an der Bildung von Knochengewebe und somit wichtig für die Struktur und Festigkeit der Knochen. Magnesium ist außerdem ein Co-Faktor von über 600 enzymatischen Reaktionen, die an verschiedenen Stoffwechselprozessen im Körper beteiligt sind [41].

Das Altern wird oftmals mit einem Magnesiummangel in Verbindung gebracht. Aufgrund physiologischer Veränderungen kommt es zu einer unzureichenden Magnesiumabsorption oder erhöhter Magnesiumausscheidung im Urin wodurch ein Defizit des Mineralstoffes entsteht. Weiterhin können Medikamente oder altersassoziierte Krankheiten und Komorbiditäten ein Magnesiumdefizit begünstigen [41]. Ein Magnesiummangel wirkt sich auf das neuromuskuläre und zentrale Nervensystem aus und kann zu Muskelkrämpfen, Zittern, Schwindel, Krämpfen und Depressionen führen. Weiterhin kann ein Magnesiummangel Krankheiten, die mit zunehmendem Alter auftreten, wie Osteoporose, atherosklerotische Gefäßerkrankungen, Herzinfarkt und Bluthochdruck, begünstigen [42]. In Deutschland liegen jeweils 34 % der Männer und Frauen über 65 Jahren unter der täglichen Zufuhrempfehlung von Magnesium [31]. Eine Portion (25 g) Cashewnüsse (258 mg/100 g) deckt bereits 1/5 des täglich erforderlichen Magnesiums ab. Eine Kombination mit weiteren magnesiumreichen Lebensmitteln, wie zum Beispiel gekochtem Naturreis (41 mg/100 g), Blattspinat (62 mg/100 g) oder Vollkornbrot (55 mg/100 g), trägt dazu bei, die tägliche Zufuhrempfehlung von Magnesium zu erreichen [43].

#### Eisen, Folat und Calcium.

Weitere nennenswerte Mikronährstoffe sind Eisen, Folat, und Calcium. Eisen ist das am häufigsten vorkommende Spurenelement im menschlichen Körper. Es ist für verschiedene Prozesse und Reaktionen im Energie- und Substratstoffwechsel notwendig. Die Hauptfunktion von Eisen besteht darin, ein funktioneller Bestandteil von Häm und von Eisen-Schwefel-Proteinen zu sein. Eisen ist somit an Sauerstoffbindung und -transport, Zellatmung und Elektronentransport beteiligt. Ein Mangel an Eisen führt zu Beeinträchtigungen der körperlichen und kognitiven Funktionen [44]. Zu den häufigsten Symptomen zählen Müdigkeit, Lethargie, Konzentrationsschwäche, Schwindel, Blässe und Kopfschmerzen [45]. Bei ersten klinischen Auswirkungen kommt es zu einer Eisen-Mangel-Anämie. Laut der KORA-Studie hatten 11 % der Teilnehmenden einen subklinischen Eisenmangel [32].

Folat, auch Vitamin B9 genannt, spielt eine wichtige Rolle bei der DNA-Synthese, -Reparatur und -Stabilität und somit bei der Produktion von Erythrozyten [44]. Ein Defizit des Vitamins im Alter kommt bei ca. 10 % der Älteren vor [32, 39] und führt zu Müdigkeit, Schlaflosigkeit und megaloblastischer Anämie sowie Depressionen und kognitiven Beeinträchtigungen [44, 46].

Calcium ist insbesondere wichtig für die Knochengesundheit im Alter. Ein lang anhaltender Calciummangel kann zu Zahnproblemen sowie zu Osteoporose führen [46].

### Mikronährstoffversorgung als Risikofaktor für Erkrankungen im Alter

Altersassoziierte Erkrankungen wie beispielsweise Alzheimer-Demenz oder Frailty werden mit verschiedenen Mikronährstoffen in Verbindung gebracht. Vitamin B12 (teilweise in Kombination mit anderen B‑Vitaminen) soll vorteilhafte Effekte auf kognitive Funktionen besitzen und Hirnatrophie bei älteren Menschen mit kognitiven Beeinträchtigungen entgegenwirken [38]. Einige Studien zeigen, dass an Alzheimer erkrankte Patienten eine geringere Vitamin-B12-Konzentration im Plasma aufweisen als ihre gesunden Kontrollgruppen [38]. Diese Befunde führen zu der Vermutung, dass ein Mangel an Vitamin B12 mit einem erhöhten Risiko für Alzheimer verbunden ist. Es gibt jedoch Studien, die zu dem Ergebnis kamen, dass ein Vitamin-B12-Mangel nicht mit einem erhöhten Alzheimer-Risiko assoziiert ist [38]. Ein suboptimaler Vitamin-D-Status wird mit geringer körperlicher Aktivität, Schwäche und Gebrechlichkeit – die Hauptmerkmale von Frailty – in Verbindung gebracht. Kochlik et al. zeigten, dass gesunde Probanden eine höhere Vitamin‑D_3_-Konzentration im Blutplasma besaßen als Probanden mit Frailty. Es ist jedoch unklar, ob eine Unterversorgung mit Vitamin D zu Frailty führt oder umgekehrt [47]. Eine suboptimale Mikronährstoffversorgung kann Erkrankungen hervorrufen. Es gibt jedoch nur wenig Evidenz über die suboptimale Mikronährstoffversorgung als Risikofaktor für altersassoziierte Erkrankungen.

### Nutzen und Risiken einer Supplementierung

Zahlreiche Studien untersuchten den Nutzen von Supplementen bei der Prävention altersassoziierter Erkrankungen, hier werden einige exemplarisch genannt. Die COSMOS-Studie untersuchte die Effekte einer 3‑jährigen Supplementation mit Kakaoextrakt vs. Placebo oder Multivitaminpräparat vs. Placebo auf kardiovaskuläre und Krebserkrankungen [48] sowie die kognitive Funktion [48, 49]. Während der Kakaoextrakt keinen Effekt auf die kognitiven Fähigkeiten hatte, wiesen Teilnehmende der Multivitamingruppe signifikante Verbesserungen auf [49]. Auch die Gedächtnisleistung wurde durch das Multivitaminpräparat nach 1 und 3 Jahren verbessert [48]. Die Ergebnisse sollten in weiteren Kohorten überprüft werden, da mit einem ähnlichen Multivitaminpräparat in der Physician’s Health Study II keine vergleichbaren Effekte gefunden wurden [50]. Die Physician’s Health Study II fand in Bezug auf altersbedingten grauen Star bei Männern, dass die mehrjährige Einnahme von Vitamin E alle 2 Tage zusammen mit der täglichen Einnahme von Vitamin C keinen Effekt auf das Risiko für grauen Star hatte [51]. Eine Metaanalyse von Placebo-kontrollierten Studien lässt keine Empfehlung für Vitamin E zur Prävention verschiedener Schlaganfalltypen zu [52].

Laut der Nationalen Verzehrsstudie II (NVS II) steigt in den höheren Altersgruppen die Nährstoffzufuhr über Supplemente bei den Vitaminen D, E und C sowie Calcium und Magnesium an [31]. Dennoch wird für Vitamin D auch durch Supplementierung die empfohlene Nährstoffzufuhr nur teilweise erreicht. Bei den B‑Vitaminen B1, B2, B6 und Niacin werden die Empfehlung hingegen oft überschritten. Für die Vitamine A, E, B12, C und Folsäure sowie für verschiedene Mineralstoffe werden die Referenzwerte selbst mit Supplementierung nur teilweise erreicht. Zu beachten ist, dass die NVS II von 2008 stammt, neuere Daten aus Deutschland gibt es in dieser Form nicht.

Es gibt einige Risiken, die es bei der Supplementierung mit Nahrungsergänzungsmitteln zu beachten gilt. Die Aufnahme von Nahrungsergänzungsmitteln kann stark variieren, was es schwierig macht, ihre Wirkung vorherzusagen. Zudem können Nahrungsergänzungsmittel mit verschreibungspflichtigen und rezeptfreien Medikamenten wechselwirken und deren Wirksamkeit beeinträchtigen oder unerwünschte Effekte verursachen [53]. Weiterhin besteht die Möglichkeit, dass Symptome von Grunderkrankungen verdeckt werden, was die Diagnose und Behandlung verzögern könnte. Da Nahrungsergänzungsmittel nicht so streng reguliert werden wie Arzneimittel, ist auch das Risiko einer Kontamination mit schädlichen Substanzen erhöht. Viele Präparate, insbesondere solche mit Magnesium und Calcium, können Magen-Darm-Probleme wie Übelkeit, Durchfall oder Verstopfung verursachen. Ein übermäßiger Calciumkonsum kann zu Hyperkalzämie führen, die Verwirrung und Herzprobleme verursachen kann. Eine Überdosierung von Vitamin D kann toxische Effekte haben. Hohe Dosen bestimmter B‑Vitamine, insbesondere B6, können Nervenschäden verursachen.

## Fazit

Das Altern ist ein fortschreitender Prozess, der den zunehmenden Verlust psychischer und physischer Funktionen umfasst. Es kommt zu Veränderungen in verschiedensten Organen und Geweben im alternden Menschen. Der Alterungsprozess und die Entwicklung altersassoziierter Erkrankungen werden unter anderem durch die Ernährung beeinflusst. Ein Mangel an Mikronährstoffen kann erhebliche negative Effekte auf die Gesundheit im Alter bewirken. Obst, Gemüse, Nüsse, Hülsenfrüchte, Milch- und Vollkornprodukte sind Quellen vieler unterschiedlicher Mikronährstoffe. Ein Verzehr dieser Nahrungsmittel hilft den Mikronährstoffbedarf im Alter zu decken und kann helfen die altersassoziierte Veränderung hinauszuzögern und somit auch Krankheiten vorzubeugen. Es wird empfohlen, die Blutwerte regelmäßig bei der/dem behandelnden Hausärztin/Hausarzt kontrollieren zu lassen, um eine mögliche Hypovitaminose zu erkennen und ihr ggf. vorzubeugen.
